# A specific fungal transcription factor controls effector gene expression and orchestrates the establishment of the necrotrophic pathogen lifestyle on wheat

**DOI:** 10.1038/s41598-019-52444-7

**Published:** 2019-11-04

**Authors:** Darcy A. B. Jones, Evan John, Kasia Rybak, Huyen T. T. Phan, Karam B. Singh, Shao-Yu Lin, Peter S. Solomon, Richard P. Oliver, Kar-Chun Tan

**Affiliations:** 1 School of Molecular and Life Sciences, Centre for Crop and Disease Management, Curtin University, Bentley, 6102 Perth, Western Australia Australia; 2 CSIRO Agriculture and Food, Wembley, Western Australia Australia; 3 Division of Plant Sciences, Research School of Biology, The Australian National University, Canberra, ACT Australia

**Keywords:** Fungal genetics, Fungal pathogenesis

## Abstract

The fungus *Parastagonospora nodorum* infects wheat through the use of necrotrophic effector (NE) proteins that cause host-specific tissue necrosis. The Zn_2_Cys_6_ transcription factor PnPf2 positively regulates NE gene expression and is required for virulence on wheat. Little is known about other downstream targets of PnPf2. We compared the transcriptomes of the *P*. *nodorum* wildtype and a strain deleted in *PnPf2* (*pf2-69*) during *in vitro* growth and host infection to further elucidate targets of PnPf2 signalling. Gene ontology enrichment analysis of the differentially expressed (DE) genes revealed that genes associated with plant cell wall degradation and proteolysis were enriched in down-regulated DE gene sets in *pf2-69* compared to SN15. In contrast, genes associated with redox control, nutrient and ion transport were up-regulated in the mutant. Further analysis of the DE gene set revealed that PnPf2 positively regulates twelve genes that encode effector-like proteins. Two of these genes encode proteins with homology to previously characterised effectors in other fungal phytopathogens. In addition to modulating effector gene expression, PnPf2 may play a broader role in the establishment of a necrotrophic lifestyle by orchestrating the expression of genes associated with plant cell wall degradation and nutrient assimilation.

## Introduction

The fungus *Parastagonospora nodorum* causes septoria nodorum blotch (SNB) of wheat. *P*. *nodorum* uses necrotrophic effectors (NEs) to cause tissue necrosis and facilitate infection of hosts possessing dominant susceptibility genes. The genes encoding three of these NEs are known: *SnToxA*, *SnTox1*, and *SnTox*3. *SnToxA* encodes a 13.2 kDa mature protein that causes necrosis on wheat cultivars that possess the dominant susceptibility gene *Tsn1*^1,2^. Near-identical copies of *ToxA* have been found in two other wheat fungal pathogens, *Pyrenophora tritici-repentis* (*Ptr*)^3^ and *Bipolaris sorokiniana*^4^. These may have been horizontally acquired, presumably from *P*. *nodorum*^1^. SnTox1 encodes a 10.3 kDa cysteine-rich mature protein that causes necrosis and confers virulence on wheat cultivars possessing *Snn1*^5^. SnTox3 is also a cysteine-rich NE. Sensitivity to the effector is conferred by either *Snn3-B1* or *Snn3-D1* located on wheat chromosomes 5BS and 5DS, respectively^6,7^. Genetic studies and protein purification assays indicate that *P*. *nodorum* possesses many more unidentified effectors associated with SNB^8^.

*SnToxA*, *SnTox1* and *SnTox3* are highly expressed during early infection but their expression is greatly decreases during saprophytic growth on the necrotised host tissue^9^. However, else was known about factors affecting their regulation until recently. Studies of TFs in *P*. *nodorum* have also provided some insights into effector gene regulation. Deletion of the APSES-class TF gene *SnStuA* in *P*. *nodorum* resulted in mutants with abnormal vegetative growth, loss of sporulation and a complete loss of virulence on wheat^10^. The expression of *SnTox3* was significantly down-regulated in the mutant, though the loss in virulence is likely attributable to pleotropic effects incurred by the mutation. A C_2_H_2_ zinc finger TF PnCon7 that binds to the promoter region of *SnTox3* was identified using a combination of yeast-1-hybrid (Y1H) and DNase footprinting, suggesting that PnCon7 may directly regulate *SnTox3* expression^11^. Silencing of *PnCon7* drastically reduced *SnTox3* expression, suggesting that PnCon7 may be a direct regulator^11^.

Cho *et al*.^12^ identified and characterised a Pleosporales-specific zinc-finger TF gene *Abpf2* from *Alternaria brassicicola* using gene knockout methods. Mutants lacking *AbPf2* were non-pathogenic on various brassica hosts. Gene expression analysis using RNAseq identified eight putative candidate effector genes that were positively regulated by AbPf2. A BLAST search of AbPf2 against the *P*. *nodorum* predicted protein set identified a conserved homolog, PnPf2^9^. Functional analysis revealed that PnPf2 is a positive regulator of *SnToxA* and *SnTox3* expression and mutants lacking *PnPf2* were only infective on *Snn1* wheat lines^9^. Based on all evidence observed, we hypothesise that PnPf2 regulates the expression of novel effectors in *P*. *nodorum*. Firstly, *P*. *nodorum* SN15 carrying *SnToxA*, *SnTox1* and *SnTox3* deletions (*toxa13*) retained the ability to produce culture filtrate that cause host-specific chlorosis^13^ and remained highly pathogenic on many modern bread wheat lines^14^. Secondly, genetic analysis revealed new quantitative trait loci for SNB were detected on wheat mapping populations^14,15^. It is possible that these QTL may be associated with novel dominant susceptibility genes^8^. Lastly, SN15 carrying deletions in both *PnPf2* and *SnTox1* lost the ability to infect all wheat lines tested including those that demonstrated susceptibility to *P*. *nodorum toxa13*^9^. This strongly suggests that PnPf2 positively regulates the expression of novel effector genes. To investigate this hypothesis and dissect other biochemical aspects of PnPf2 regulation, we used RNAseq to compare the gene expression profiles of a *P*. *nodorum pnpf2* mutant with the wildtype strain under conditions that are conducive for effector gene expression.

## Results

### *PnPf2* is required for full hyphal proliferation during host infection

**Figure 1:**
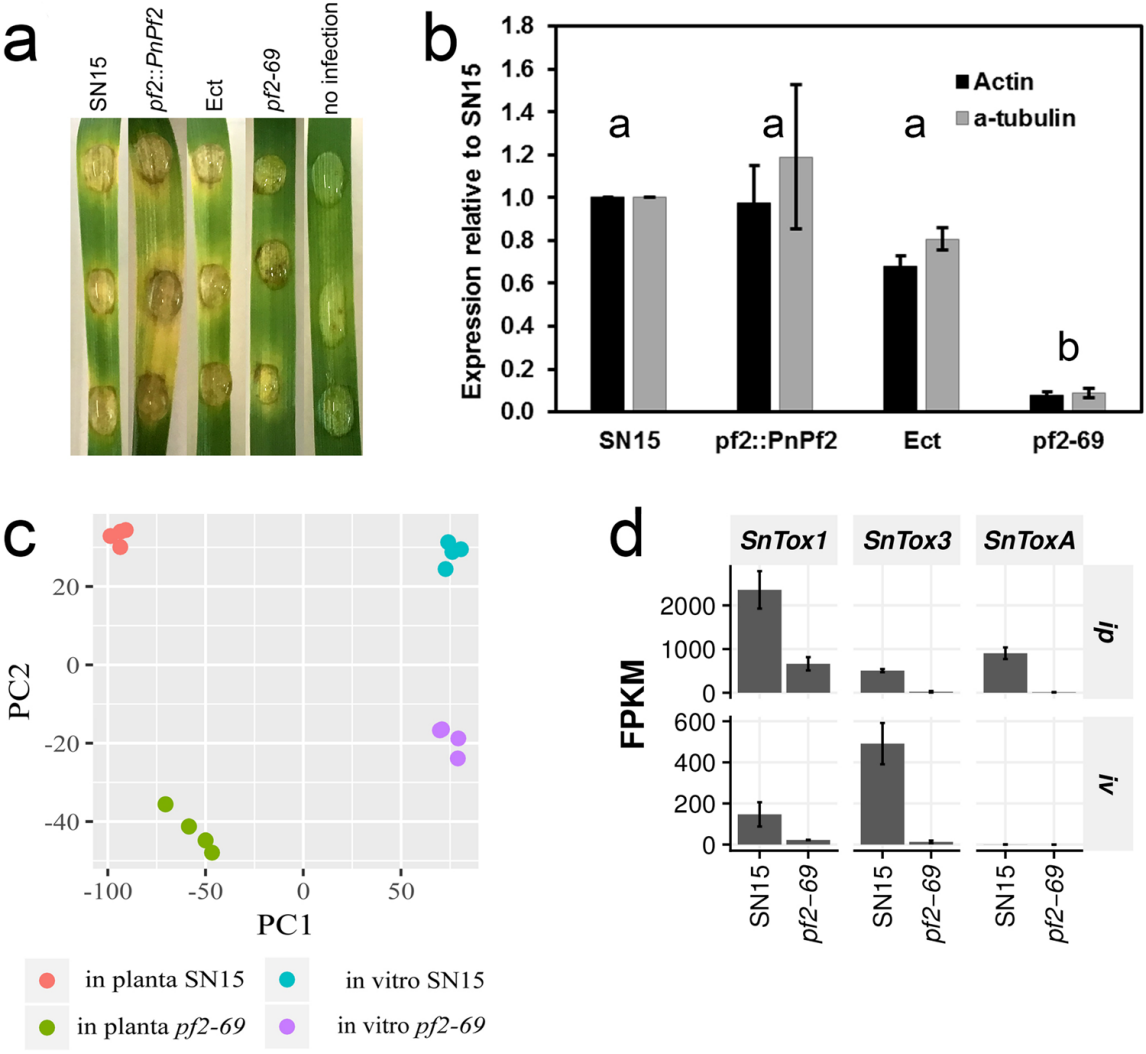
Infection, biomass and RNAseq analysis. (**a**) The onset of chlorotic symptoms was observed for SN15, *pf2-69*, Ect and *pf2*::*PnPf2*-infected wheat at three dpi. (**b**) Q-PCR quantification of biomass via fungal gDNA. Average biomass levels determined from PCR amplification of actin and α-tubulin not connected by the same letter are significantly different (*P* < 0.05) based on ANOVA (*n* = 3). (**c**) Comparing transcriptomes of SN15 and *pf2-*69 sampled *in vitro* and *in planta* using PCA from DESeq. 2 normalised reads (Supplementary Data S2). PnPf2 plays a bigger regulatory role during infection compared to *in vitro* growth. PC1 and PC2 explains 71% and 12% of the total variance, respectively. (**d**) Comparative RNAseq expression profiling of *SnToxA*, *SnTox1* and *SnTox3* in SN15 and *pf2-69* under *in vitro* (*iv*) and *in planta* (*ip*) conditions. Bars show mean FPKM estimated by Cufflinks for each sample (*n* = 4), and error bars indicate standard deviation. Expression of all three effectors was reduced in the *in planta pf2-69* samples compared with SN15.

The transcriptome of the *P*. *nodorum* reference wildtype strain SN15 was compared to the *PnPf2*-deleted strain *pf2-69* grown under two conditions. Firstly, we sampled RNA during early infection at three days *in planta* (*ip*) where *PnPf2*, *SnToxA*, *SnTox1* and *SnTox3* are maximally expressed. Wheat cv. Halberd (*Tsn1*, *Snn1*, *Snn3*) was used as a host as it is susceptible to SN15 and *pf2-69*^9^. Secondly, SN15 and *pf2-69* were grown for three days *in vitro* (*iv*) in Fries 3 broth which is conducive for SnTox1 and SnTox3 production^9^. Vegetative growth of *pf2-69* was comparable to SN15^9^. Paired-end Illumina HiSeq technology was used as an RNAseq sequencing platform. The latest SN15 genome revision produced 13,563 predicted genes^16^. Deep sequencing produced more than 90% fungal transcripts that aligned to predicted genes for all samples (Supplementary Data S1 and Table 1). *In vitro* and *ip* samples returned an average of 24 million and 290 million read pair fragments (including plant reads), respectively. Between 18 and 22 million read pairs, representing an average of 6.94% of the total reads, aligned to the SN15 genome for the SN15 *ip* treatment (Supplementary Table S1). Between 3.4 and 5.9 million reads (average 1.57% of total) from *pf2-69 ip* growth aligned to the SN15 genome. The low proportion of fungal reads from *pf2-69* suggests reduced biomass during infection. Quantitative PCR of genomic DNA extracted from three days post infected wheat cv. Halberd confirmed that the biomass of *pf2-69* was significantly lower than strains carrying a functional copy of *PnPf2* (Fig. 1a,b).

**Table 1:** A functional summary of PnPf2-regulated candidate effector genes and their status in *P*. *nodorum* SN19-1087.

SN15 gene	PhiBase	Functional prediction	Size (kDa)	Length (aa)	SN79-1087 gene	Mutations (aa)	Notes
SNOG_01146	Homolog of *MoCDIP4* effector.	Cleavage of cellulose chains. CAZy family AA9 (formerly GH61)	23.5	229	03796-RA	D28E	—
SNOG_02755	—	Family with unknown function. Members in pathogens and non-pathogens. Incl biotrophs and necrotrophs.	41.6	409	02992-RA	GGQNNGQGQNNGQ31G, QNN82Q, G313GN	Repeat motif copy number variation
SNOG_02980	—	SGNH hydrolase-type esterase. Possible lipase or pectinase.	25.9	247	02810-RA	—	—
SNOG_08150	—	—	14.1	131	01518-RA	F3S, S131W	
SNOG_10736	—	—	48.8	522	10887-RA	N187NANAGNNANANAG, GANAGNNANAGAAAGNAAGNNANAGN244G, NANAG280N, GNN300G, G342GN	Repeat motif copy number variation
SNOG_12350	—	—	10.8	109	12820-RA	—	
SNOG_13939	—	Family with unknown function. Members in other pathogens. Incl biotrophs and necrotrophs	17.5	171	06645-RA	T24P, V67I, A104AAQVSISPSLTVTMMMWRNSSADAC	Intron splice site SNP in SN79-1087 creates large insertion
SNOG_14243	—	SGNH hydrolase-type esterase. Possible acetyl xylan esterase.	25.8	246	09528-RA	—	—
SNOG_15270	Homolog of *Xyn11A*	Xylanase. CAZy family GH11.	25.3	231	04223-RA	—	Numerous paralogs.
SNOG_30077	—	—	7.1	66	03763-RA	F39A, P49L, S60A, RACC63VSSRESRMRVDTILMLLYSALAAHLVPVPKVGV	SNP interrupts stop codon in SN79-1087, extended protein.
SNOG_30352	—	—	8.4	79	07626-RA	A12T	
SNOG_30359	—	—	8.3	76	07575-RA	—	—

### Analysis of differentially expressed (DE) genes

Genes were considered DE in a contrast of isolate or treatment if tests of absolute log_2_ fold change >1 were consistently significant (*P*_adj_ < 0.05) for three test methods described below (Supplementary Data S3). Because *pf2-69 ip* samples had considerably fewer reads than other samples, an additional filter requiring *pf2-69 ip* samples to have > = 10 counts per million (CPM) for a gene to be called down-regulated was used for high-confidence DE prediction sets. For SN15 *ip* and *iv* treatments, 1,889 genes were up-regulated and 1393 were down-regulated *ip* (Supplementary Table S2). A total of 1,736 genes were up-regulated and 706 genes were down-regulated between the *pf2-69 ip* and *iv* treatments. For *ip* comparisons, 303 genes were significantly reduced whereas 449 were up-regulated in *pf2-69* over SN15. Additional DE genes were observed using relaxed criteria, allowing genes where any of the three tests are significant (<3 tests) (Supplementary Table S2). The main difference between the three tests results were in how they handle contrasts involving samples with few or no reads aligned to the gene. Additional genes involving *pf2-69 ip* samples with <10 CPM were identified using the same relaxed criteria. In total, 269 gene were down-regulated in *pf2-69 ip* compared to *iv* growth and had fewer than 10 CPM in *pf2-69* during *ip* growth. Similarly, 163 genes were down-regulated in *pf2-69* during infection compared to SN15 and had fewer than 10 CPM in *pf2-69* during *ip* growth (Supplementary Table S2).

A principal component analysis (PCA) plot for PC1 and PC2 was constructed based on normalised fragment counts per gene to describe the variation between and within each treatment (Fig. 1c). The biological replicates tightly clustered together, with each treatment strongly differentiated from the others. This indicates that sample treatment and sequencing did not contribute to systematic biases that could not be removed by normalisation. PC1 captured 71% of the total variance and discriminated *iv* from *ip* samples. PC2 captured 12% of the variance and discriminated SN15 from *pf2-69*.

We then examined *SnToxA*, *SnTox1* and *SnTox3* expression profiles (Fig. 1d). As expected, the expression of SnToxA and SnTox3 was almost abolished in *pf2-69 ip*. SnTox3 expression was also highly reduced in *pf2-69 iv*. *SnToxA* is poorly expressed in SN15 and *pf2-69* during *iv* growth. *SnTox1* expression was significantly higher in SN15 compared to *pf2-69*. *SnTox1* is still strongly expressed during *ip* growth and had the lowest fold change difference between SN15 and *pf2-69 ip* compared to *SnToxA* and *SnTox3*.

### PnPf2 regulates genes that encode effector-like proteins

**Figure 2:**
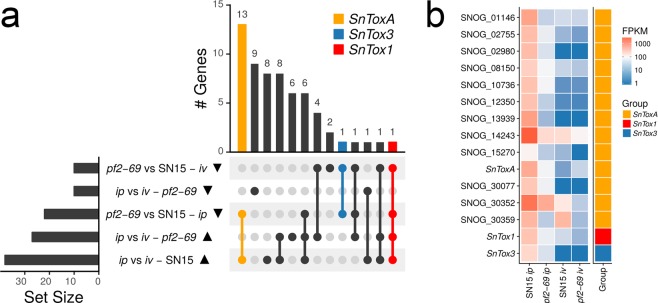
Identification of SN15 candidate effector genes positively regulated by PnPf2. (**a**) An UpSet plot demonstrating the number of candidate effector genes that displayed similar expression profiles. Rows in the matrix represents sets of differentially expressed effector candidates in a contrast, with the solid triangles indicating the direction of expression change. Lines connecting rows of the matrix indicate an intersection between the sets, and the vertical bar chart indicates the number of genes that are common to those sets. Set intersections containing known effectors are indicated with colour. (**b**) A heatmap showing mean FPKM (*n* = 4) profiles of the candidate effector genes that share a common expression profile with *SnToxA*.

To identify candidate effector genes positively regulated by PnPf2, we analysed genes that were down-regulated in *pf2-69* that possessed a secretory signal peptide (but no transmembrane domains outside of the signal peptide) and were predicted to be effector-like by EffectorP^17^. Twelve genes that showed a similar expression profile to *SnToxA* (ie. down-regulated in *pf2-69 ip* compared to SN15 *ip* and up-regulated *ip* in both strains) were identified (Fig. 2). In contrast, *SnTox1* and *SnTox3* were the only effector genes categorised in their respective expression profile categories (Fig. 2). The expression profiles of these candidate effector genes in SN15 and *pf2-69* three days post-infection were validated using qRT-PCR (Supplementary Fig. S1). Apart from SNOG_10736, SNOG_13939 and SNOG_02980, the qRT-PCR-based expression profile of all other candidate effector genes between SN15 and *pf2-69* was consistent with findings from the RNAseq data. The expression profiles of the 12 candidate effector genes in SN15 were examined between three and 10 days post-infection using available microarray gene expression data^18^ and qRT-PCR analyses performed in this study (Supplementary Fig. S2). SNOG_08150, SNOG_13939, SNOG_30077, SNOG_30352 and SNOG_30359 demonstrated similar expression profiles to *SnToxA*, *SnTox1* and *SnTox3* where gene expression peaked at three dpi and decreased to almost non-detectable levels at seven and 10 dpi, coinciding with host tissue necrosis.

Four of the 12 candidate effectors possess Pfam domains (Supplementary Data S4 and Table 1). SNOG_01146 and SNOG_15270 possess a glycosyl hydrolase family domain. SNOG_02980 and SNOG_14243 both possess a hydrolase-type esterase family domain. A BLAST search of PHIbase^19^ indicated that SNOG_01146 displays significant amino acid sequence similarities to MoCDIP4 (*Magnaporthe oryzae* cell death–inducing protein P4) of the rice blast fungus *M*. *oryzae*^20^ whereas SNOG_15270 is similar to the *Botrytis cinerea* partial virulence determinant gene *Xyn11A* which encodes a xylanase^21^. Pfam domains were not observed for the other six candidates (Supplementary Data S4 and Table 1) but SNOG_08150, 12350, 30352, 30359 and 30077 encode small cysteine-rich (<20 kDa) proteins and BlastP analyses of SNOG_02755. 08150, 10736, 12350 and 13939 revealed significant hits to other fungal hypothetical proteins, whereas SNOG_30352, 30359 and 30077 appear to be unique to *P*. *nodorum* based on tBlastN searches.

*P*. *nodorum* SN79-1087 is non-pathogenic on wheat and lacks *SnToxA*, *1* and *3*^5^. We decided to investigate if these 12 candidate effectors are present or altered in SN79-1087^22^. BlastP and tBlastN analysis revealed five genes were identical between SN15 and SN79-1087. SNOG_02755 and 10736 are also present in SN79-1087, but both have in-frame deletions in low-complexity amino acid repeat regions. Changes in amino acid sequence were observed for seven gene homologs in SN79-1087 (Table 1). Frame shifts or premature stop codons were not observed for these genes.

### PnPf2 regulates depolymerase and nutrient assimilation gene expression *in planta*

**Figure 3:**
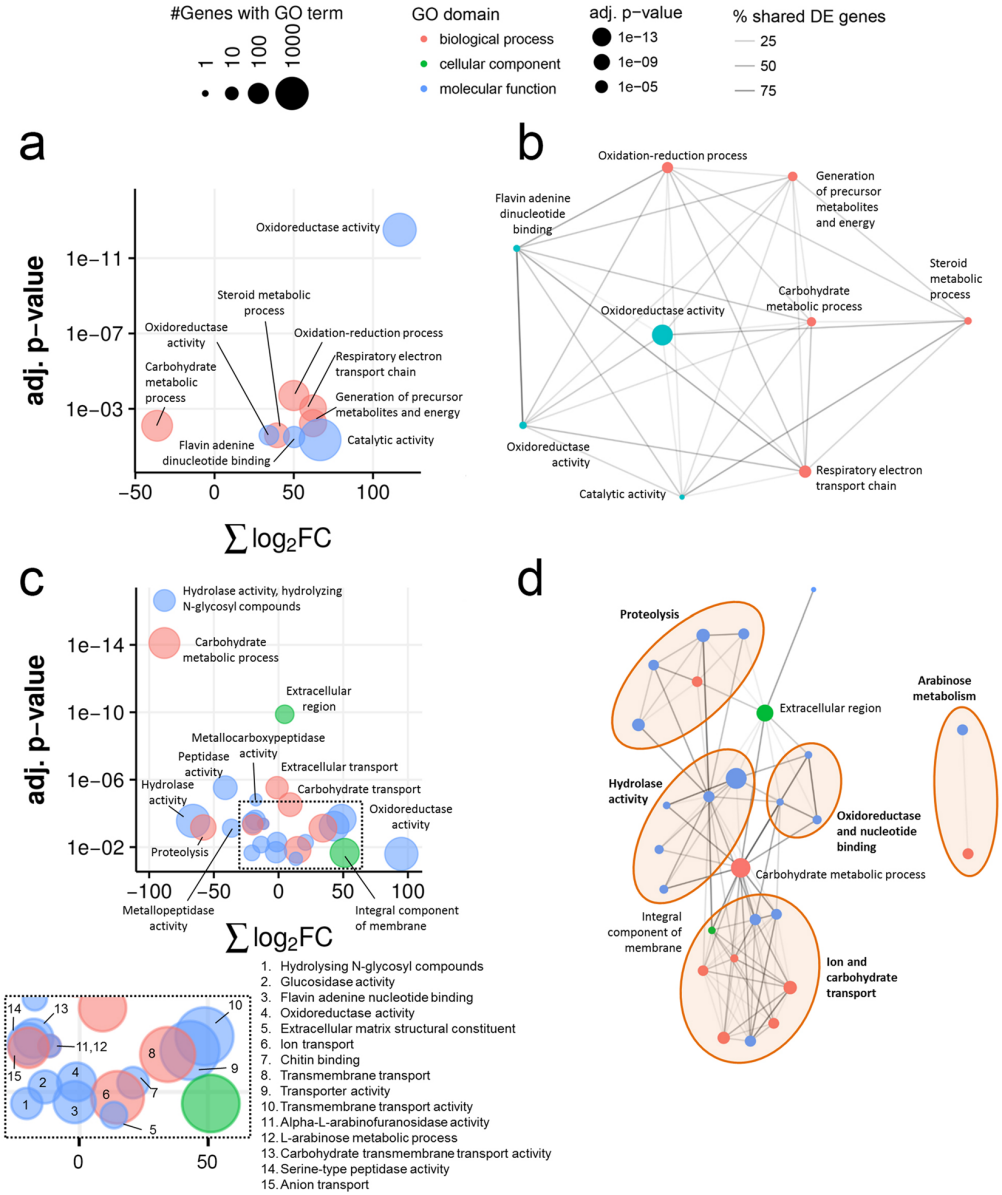
An illustrated summary of gene ontology (GO) term enrichment analysis between *pf2-69* and SN15 *in vitro* (**a**,**b**) and *in planta* (c and d). Bubble plots illustrate GO terms that were over-represented in differentially expressed (DE) genes as the sum of all log_2_ fold changes of *pf2-69* relative to SN15 and the statistical significance of enrichment tests for GO terms (**a**,**c**). Bubble size indicates the number of genes annotated with that GO term. The relationships between significantly over-represented GO terms were highlighted using network analysis (**b**,**d**). Nodes represent a single GO term and are connected if a DE gene is annotated with both terms, with the shade of the edges indicating the proportion of genes with both GO terms that are differentially expressed. Node sizes indicate the statistical significance of GO term enrichment tests. Detailed GO term analysis is deposited as Supplementary Data S5. Interactive GO enrichment and network plots are deposited as Supplementary Data S6.

To investigate changes in overall biochemical processes between SN15 and *pf2-69* during *iv* and *ip* growth, we assessed DE genes for enrichment of GO terms^23^ (Fig. 3). GO terms were assigned to all genes where possible using InterProScan^24^ and dbCAN^25^.

During *iv* growth, genes categorised under oxidoreductase activities, flavin adenine dinucleotide binding and catalytic activity were significantly up-regulated in *pf2-69* (Fig. 3a). The majority of these genes encode cytochrome P450s, FAD binding proteins and oxidases (Supplementary Data S3 and S5). GO network analysis revealed that differentially expressed genes associated with oxidoreductase activities are central to biological processes related to respiratory electron transport chain, steroid metabolism, redox, carbohydrate metabolism and generation of precursor metabolites and energy (Fig. 3b).

During *ip* growth, molecular functions (MFs) associated with hydrolase, glucosidase and peptidase activities tended to be down-regulated in *pf2-69* (Fig. 3c). The MF hydrolase activity associated with hydrolysing N-glycosyl compounds consisted of 155 genes. Of these, the expression of 39 genes were significantly lower in *pf2-69*. Similarly, the MF associated with another hydrolase activity associated with hydrolysing N-glycosyl compounds consisted of 32 genes, of which eight genes were significantly down-regulated in *pf2-69* compared to SN15. The MF hydrolase activity consisted of 1,168 genes. Of these, 64 were expressed at lower levels in *pf2-69*. The majority of genes annotated encode plant cell wall degrading enzymes (CWDEs) and other carbohydrate depolymerases such as β-xylosidases, acetyl xylan esterases, glucanases and glucosidases (Supplementary Data S3 & S5). Arabinose is a major constituent of the plant cell wall. GO enrichment indicates that PnPf2 regulates arabinose metabolism in *P*. *nodorum*. Of the six genes associated with α-L-arabinofuranosidase activity, five were expressed at lower levels in *pf2-69* (Fig. 3c,d).

For protein degradation, 240 genes encode proteins with predicted peptidase activity were differentially expressed (GO:0008233) (Fig. 3c). Of these, 29 were down-regulated in *pf2-69* compared to SN15. Additionally, 63 genes encoding proteins with putative metallopeptidase activity were identified from the genome. Of these, 14 were down-regulated in *pf2-69 ip* compared to SN15. The MF associated with metallocarboxypeptidase activity (GO:0004181) consisted of nine genes where the expression of seven was reduced in *pf2-69*. For the MF associated with serine-type peptidase activity, 22 of 131 genes were expressed at lower levels in *pf2-69*. CAZyme and Interpro analyses of genes classified under GO:0008233, 0004181, 0008237 and 0008236 indicate that most encode peptidases and esterases (Supplementary Data S3 and S5).

GO analysis revealed that cellular redox potential in *pf2-69* was perturbed during *ip* growth in addition to a similar defect observed during *iv* growth. A MF associated with oxidoreductase (GO:0016491) activity was enriched in up-regulated genes in *pf2-69 ip* compared to SN15 (Fig. 3c). The majority of DE genes encoding oxidases, cytochrome P450s, reductases and dehydrogenases (Supplementary Data S3 and S5) are associated with a biological role in carbohydrate metabolism (Fig. 3d). In addition, MFs linked to transport activities were enriched with genes that were similarly up-regulated in *pf2-69 ip* compared to SN15 (Fig. 3c). Genes associated with the transport function encode sugar and amino acid transporters (Fig. 3d; Supplementary Data S3 and S5).

### Identification of DNA motifs enriched in the promoters of PnPf2-regulated genes

**Figure 4:**
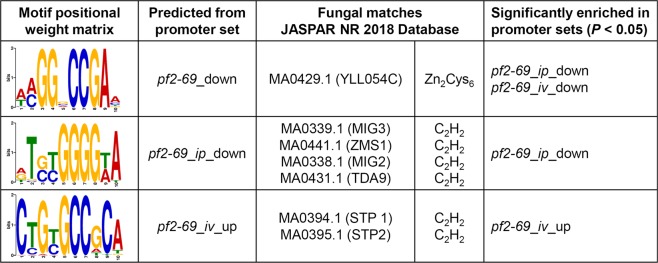
Identification of motifs displaying enrichment in promoters of DE genes. Promoter sets used to model the motifs are listed in the second column. The third column contains motif matches to known fungal TFBSs in the JASPAR 2018 non-redundant database and their associated TF family. The fourth column lists the treatment groups displaying enrichment of the respective motif in the promoter set. Motif frequency and statistical analysis are described in Supplementary Data S7.

We hypothesised that a shared *pf2-69* DE patterns implied a common transcriptional regulator. Therefore, promoters of these gene sets may harbor over-represented motif(s) functioning as potential *PnPf2* transcription factor binding site(s) (TFBS). Analysis of the promoters from the respective *pf2-69* DE gene groupings revealed three such motifs (Fig. 4). The motif WMGGVCCGAA, enriched in *pf2-69 iv* and *ip* down-regulated gene promoters, is similar to an enriched motif associated with AbPf2 down-regulated genes in *A*. *brassicicola*^12^ and is characteristic of a Zn_2_Cys_6_ TFBS^26,27^. A second motif resembling a C_2_H_2_ TFBS (RTSYGGGGWA) was significantly enriched in *pf2-69 ip* down-regulated gene promoters. The third motif (CTGYGCCGCA) also resembled a C_2_H_2_ TFBS and was enriched in *pf2-69 iv* up-regulated gene promoters. The identification of unique enriched motifs in the separate datasets suggests that PnPf2 may act as an indirect regulator or its binding site specificity can be influenced by other regulators of target genes.

### Absence of interaction between PnPf2 and the putative consensus motif on *SnToxA* and *SnTox3* promoters

**Figure 5:**
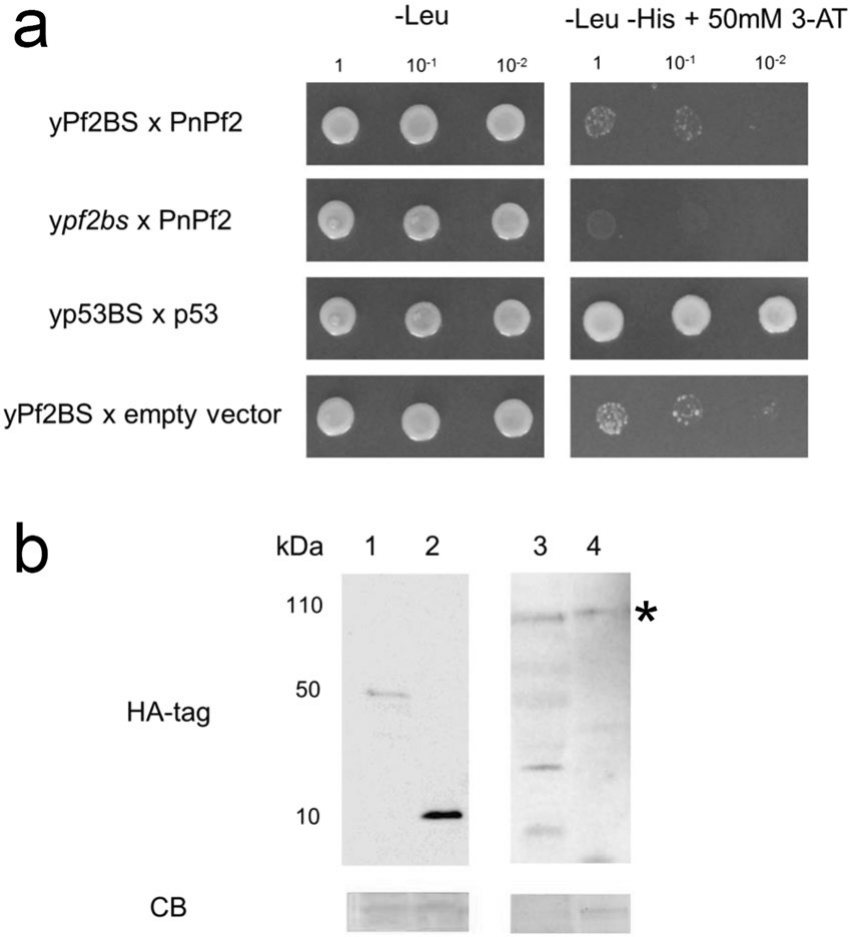
Y1H analysis of PnPf2 and putative promoter motif interaction. (**a**) yPf2BS expressing PnPf2 grew on the -Leu medium; however, was not able to grow on the -Leu -His medium. 50 mM 3-AT was added to the -Leu -His plate for preventing possible histidine leakage. Mutated PnPf2 binding site (pf2bs) and empty vector were manipulated as negative controls. *p53* interaction was used as positive control. Dilutions of yeast cells are indicated. (**b**) Western blots using HA antibody and coomassie blue (CB) staining of yeast cell extracts. 1: yp53BS x p53. 2: yPf2BS x empty vector. 3: yPf2BS x PnPf2. 4: ypf2bs x PnPf2. The PnPf2-GAL4AD-HA tag protein band is indicated (*). These are cropped images from different gel and blot photos. Original photos are supplied as Supplementary Fig. S3 where possible.

Inspection of the *SnToxA* and *SnTox3* promoter region revealed at least one occurrence of the WMGGVCCGAA motif consensus sequences that was absent from *SnTox1*. For *SnToxA*, the consensus sequence was identified at 218, 364 and 416 bp upstream of the transcriptional start site. The consensus sequence was also observed at two sites in the *PtrToxA* promoter of *Ptr*. For *SnTox3*, the consensus sequence was identified at 679 bp upstream of the transcriptional start site. This consensus sequence was not observed in the promoter region of *SnTox1*. Therefore, it was hypothesised that WMGGVCCGAA functions as a PnPf2 binding site (Pf2BS). A yeast 1-hybrid (Y1H) assay was performed in order to determine whether PnPf2 can directly interact with the putative binding site represented in the *SnToxA* promoter. No significant interaction was observed between PnPf2 and four tandem repeats of the Pf2BS (Fig. 5a). Western blot analysis confirmed the presence of the PnPf2 protein indicating that the absence of Y1H interaction was not the result of the lack of protein (Fig. 5b).

### Identification of DE TF genes

We then screened for putative TF genes that were DE between SN15 and *pf2-69* from the high confidence DE gene set to explore the possibility that PnPf2 operates indirectly. We limited our search to genes that encode proteins with TF domains found in fungi^28^. A total of 20 DE putative TFs were identified covering both *iv* and *ip* treatments. Based on distinct InterPro classifications^24^, this set consisted of five basic leucine zippers, one zinc knuckle, one myc-type, one CCHC-type, one p53-like, one C_2_H_2_, one homeodomain-like, six fungal specific Zn_2_Cys_6_ and three unspecified fungal TFs (Table 2). A BLAST search of PHIbase^19^ revealed that seven of these DE TF genes have strong matches to other fungal TFs associated with virulence (Table 2). Three of these belong to the fungal-specific Zn_2_Cys_6_ class (SNOG_03490, 07307 and 08440), one homeodomain-like (SNOG_08237) and three basic-leucine zippers (SNOG_04486, 13689 and 16487).

**Table 2:** A description of DE putative *P*. *nodorum* TF genes, domains and amino acid (aa) identity to characterised orthologs in other fungal pathogens.

Gene	*pf2-69* vs SN15 (*ip*)	*pf2-69* vs SN15 (*iv*)	Interpro description	Top PHI-BLAST gene hit (Pathogen*)	Mutant phenotype	E value (% aa identity)	Reference
SNOG_00166	same	down	Basic-leucine zipper domain	GzbZIP020 (*Fg*)	Unaffected pathogenicity	3.34E-52 (50)	Son, *et al*.^40^
SNOG_00439	down	same	Transcription factor domain, fungi	GzZC252 (*Fg*)	Unaffected pathogenicity	1E-150 (42)	Son, *et al*.^40^
SNOG_03490	same	down	Zn_2_C_6_ fungal-type DNA-binding domain	GzZC232 (*Fg*)	Reduced virulence	0 (52)	Son, *et al*.^40^
—	—	—	—	*MoPRO1* (*Mo*)	Unaffected pathogenicity	0 (52)	Lu, *et al*.^79^
—	—	—	—	*ProA* (*Ef*)	Hypervirulence	0 (45)	Tanaka, *et al*.^80^
SNOG_04486	same	down	Basic leucine zipper domain	GzbZIP001 (*Fg*)	Reduced virulence	8.3E-118 (40)	Son, *et al*.^40^
SNOG_05500	up	same	Zinc knuckle CX2CX4HX4C	GzCCHC008 (*Fg*)	Unaffected pathogenicity	1.62E-98 (40)	Son, *et al*.^40^
SNOG_06105	same	up	Transcription factor domain	GzZC238 (*Fg*)	Unaffected pathogenicity	7.1E-139 (45)	Son, *et al*.^40^
SNOG_07070	same	up	Zn_2_C_6_ fungal-type DNA-binding domain	GzZC211 (*Fg*)	Unaffected pathogenicity	3.11E-63 (28)	Son, *et al*.^40^
SNOG_07307	same	up	Zn_2_C_6_ fungal-type DNA-binding domain	*Cca1* (*Mo*)	Loss of pathogenicity	1.2E-23 (53)	Lu, *et al*.^79^
SNOG_07556	up	same	Myc-type, basic helix-loop-helix (bHLH) domain	GzbHLH014 (*Fg*)	Unaffected pathogenicity	2.06E-08 (36)	Son, *et al*.^40^
SNOG_08237	down	same	Homeodomain-like	*MoHox5* (*Mo*)	Reduced virulence	1.23E-79 (57)	Kim, *et al*.^81^
—	—	—	—	GzHOME004 (*Fg*)	Unaffected pathogenicity	2.21E-64 (51)	Son, *et al*.^40^
SNOG_08440	same	down	Zn_2_C_6_ fungal-type DNA-binding domain	*AtrR* (*Af*)	Reduced virulence	4.9E-34 (25)	Hagiwara, *et al*.^41^
SNOG_08565	up	same	Zn_2_C_6_ fungal-type DNA-binding domain	GzZC243 (*Fg*)	Unaffected pathogenicity	1.1E-117 (39)	Son, *et al*.^40^
SNOG_11322	up	same	Zinc finger, CCHC-type	GzCCHC008 (*Fg*)	Unaffected pathogenicity	4.71E-33 (39)	Son, *et al*.^40^
SNOG_12086	up	same	Zn_2_C_6_ fungal-type DNA-binding domain	FZC87 (*Mo*)	Unaffected pathogenicity	1.09E-25 (45)	Son, *et al*.^40^
SNOG_12740	same	up	p53-like transcription factor	GzP53L005 (*Fg*)	Unaffected pathogenicity	1.88E-29 (43)	Son, *et al*.^40^
SNOG_13359	up	up	Basic-leucine zipper domain	*atfD* (*Af*)	Unaffected pathogenicity	1.47E-10 (35)	Pereira Silva *et al*.^82^
SNOG_13689	up	up	Basic-leucine zipper domain	*CgAP1* (*Cg*)	Loss of pathogenicity	2.97E-10 (47)	Li *et al*.^83^
SNOG_15627	same	up	Zinc finger, C_2_H_2_	GzC2H091 (*Fg*)	Unaffected pathogenicity	1.15E-17 (27)	Son, *et al*.^40^
SNOG_16487	up	same	Basic-leucine zipper domain	GzbZIP007 (*Fg*)	Reduced virulence	2.05E-14 (56)	Son, *et al*.^40^
SNOG_30247	up	same	Transcription factor domain, fungi	GzZC239 (*Fg*)	Unaffected pathogenicity	2.51E-17 (23)	Son, *et al*.^40^

*Fg, *Fusarium graminearum*; *Mo*, *Magnaporthe oryzae*; *Af*, *Aspergillus fumigatus*; *Ef*, *Epichloe festucae*; *Cg*, *Colletotrichum gloeosporioides.*

## Discussion

Regulation of downstream target genes including those that encode effector-like proteins by members of the Pf2 Zn_2_Cys_6_ family was first reported in Cho *et al*.^12^ in *A*. *brassisicola*. The comparative RNAseq approach employed in that study derived from *A*. *brassicicola*-infected *A*. *thaliana* tissue which yielded a total of 8.5 to 9.3 × 10^5^ reads from the WT and *abpf2* mutant sample (approximately 0.5% of total reads) that mapped to the *A*. *brassicola* genome, respectively. Much higher fungal read counts were obtained in this study through the use of deep sequencing across four biological replicates resulting in more read information to exhaustively identify DE genes between SN15 and *pf2-69* during *ip* growth (Supplementary Table S1).

RNAseq confirmed *SnToxA* and *SnTox3* down-regulation in *pf2-69* but the expression of *SnTox1* was significantly higher in SN15 than *pf2-69* than our previous observation^9^. The possibility that PnPf2 plays a minor regulatory role in *SnTox1* regulation requires further investigation. Culture filtrates derived from *pnpf2* mutants caused chlorosis on *Snn1* wheat lines although the symptom was slightly weaker than with SN15^9^. Nevertheless, *SnTox1* is still strongly expressed in *pf2-69* during infection and is sufficient to produce detectable SnTox1 activity in the culture filtrate and confer virulence on *Snn1* wheat lines^9^.

It is not known if *A*. *brassicicola* uses effectors to modulate host infection. However, Cho *et al*.^12^ identified eight genes that encode small-secreted proteins with effector-like hallmarks positively regulated by AbPf2. Candidate effector genes were identified in this study that showed the same differential expression patterns as *SnToxA* and have effector-like properties. One of the effector candidates displayed significant sequence similarities to a known effector and pathogenicity factor. SNOG_01146 possesses a glycosyl hydrolase 61 domain and showed amino acid similarity to MoCDIP4. MoCDIP4 was identified as an apoplastic effector secreted by *M*. *oryzae* that causes cell death in rice^20^. Moreover, MoCDIP4 also induces cell death in non-host eudicots. In addition, these effectors are small, cysteine rich and expressed highly during early infection. SNOG_15270 is an homolog of *Xyn11A* which encodes an endo-ß-1,4-xylanase in *B*. *cinerea*. Deletion of *Xyn11A* in *B*. *cinerea* caused a significant reduction in virulence and growth on xylan^21^. All 12 candidate effector genes are also present in SN79-1087. Seven of these candidate proteins encode altered protein sequences in SN79-1087, which may explain some difference in pathogenicity. Five proteins possess changes in amino acid residues. It was previously observed that ToxA isoforms differ greatly in necrosis-inducing activities on *Tsn1* wheats and affect the speed of asexual sporulation^29^. It is interesting to note that SNOG_02755 and 10736 polypeptides contain short amino acid sequence repeats that are partially deleted in SN79-1087. Several well-studied fungal and oomycete effectors contain repeats that possess functional roles in cellular localisation, host recognition and plant cell wall binding^30^. Additionally, recent studies have indicated that differential expression of effector genes between *P*. *nodorum* isolates affect their contributions to SNB of wheat^15,31,32^. The expression of these candidate genes in SN79-1087 requires further study.

GO enrichment revealed that PnPf2 functions as a positive regulator of a large subset of plant CWDEs and proteases during infection. Additionally, the removal of *PnPf2* resulted in a general up-regulation in expression of nutrient transporter genes during infection. It is still not known whether this change is caused directly by the absence of PnPf2, or indirectly via another mechanism regulated by PnPf2. Comparative transcriptomic analysis of *A*. *brassicicola* identified only 13 genes that encode hydrolytic enzymes including two pectate lyases, were regulated by AbPf2^12^. Deep sequencing used in this study provided a higher resolution insight into CAZyme regulation exerted by the Pf2 Zn_2_Cys_6_ class. Quantifying the contributions of plant CWDEs to phytopathogenicity is difficult because many fungal phytopathogens possess expanded gene families that result in functional redundancies^33^. For example, early studies on the causal agent of northern leaf spot of maize *Cochliobolus carbonum* (eg.^34–37^) did not find a clear role for CWDEs in fungal virulence. This is not to imply that CWDEs are dispensable for fungal virulence. It was reported that feruloyl esterases from *Valsa mali*^38^, a AbPf2-regulated pectate lyase from *A*. *brassicicola*^39^ and an endo-β-1,4-xylanase from *B*. *cinerea*^21^ function as virulence factors. Since plant CWDEs deconstruct the plant cell wall and liberate simple carbohydrates for assimilation and growth, it remains to be determined if SN15 can outcompete *pf2-69* during co-infection on *Snn1* wheats as the former can express a much larger repertoire of extracellular hydrolytic enzymes. RNAseq read counts suggested that *pf2-69* accumulated much less biomass than SN15 at three dpi. This is surprising as *pf2-69* retained the ability to causes lesions on *Snn1* wheat lines comparable to SN15 as previously observed^9^. It is probable that SnTox1 secreted by *pf2-69* during infection is the main cause of necrosis rather than the accumulation of fungal biomass at the lesion.

Analysis of the *pf2-69* DE gene sets identified three distinct over-represented motifs (Fig. 4). The most notable of these is the WMGGVCCGAA motif associated with genes under PnPf2 positive regulation, as this motif was observed at multiple sites along the *Sn*/*PtrToxA* and *SnTox3* promoters and also enriched in AbPf2-regulated gene promoters^12^. We hypothesised that it functions as a PnPf2 binding site as it resembles a Zn_2_Cys_6_ TFBS^26,27^. However, Y1H assay indicated that PnPf2 did not bind to the motif. This suggests either PnPf2 does not function as a direct regulator of *SnToxA*, *SnTox3* and DE genes through interaction with the WMGGVCCGAA motif, or that necessary PnPf2 post-translational modifications/interactions are not compatible with the Y1H system. It was noted however that six other Zn_2_Cys_6_-type TF genes were differentially expressed between *pf2-69* and SN15 (Table 2). Of these, only two were down-regulated but may serve as alternate candidates for direct regulation targeting the WMGGVCCGAA motif. A BLAST search of these against PHIbase revealed pathogenicity-associated functions in fungal homologues. SNOG_03490 is 52% identical to GzZC232 of *Fusarium graminearum*, the causal agent of fusarium head blight of wheat and is required for full virulence^40^. SNOG_08440 is homologous to a Zn_2_Cys_6_-type TF gene *AtrR* of *Aspergillus fumigatus*, an opportunistic fungal pathogen of mammals^41^. AtrR is a regulator of ergosterol biosynthesis pathway genes most notably *Cyp51*, a target for fungicide control. Deletion of *AtrR* resulted in impaired fungal growth and attenuated virulence on mice^41^. The other enriched motifs were characteristic of C_2_H_2_ binding sites^27^ however, only one DE TF of this class was identified - SNOG_15627 (Table 2). SNOG_15627 expression was up-regulated in *pf2-69* under *iv* condition but remained unchanged during *ip* growth. SNOG_15627 demonstrated weak similarity to a characterised TF in *F*. *graminearum* shown to be dispensable for pathogenicity on wheat^40^. As the CTGYGCCGCA motif was enriched in the *pf2-69 iv* up-regulated gene promoters, it is possible that SNOG_15627 functions as a direct regulator. PnCon7 is the only characterised C_2_H_2_ TF in *P*. *nodorum* involved in SnTox3-mediated disease and direct regulation^11^. However, the cis-regulatory element of PnCon7 differs to both predicted C_2_H_2_ binding sites observed in this study.

**Figure 6:**
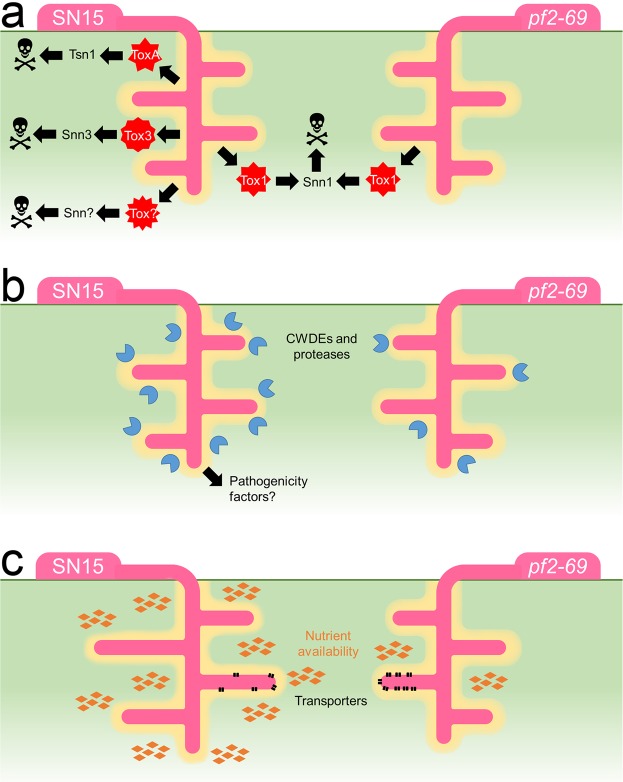
The proposed model for the role of PnPf2 during infection. (**a**) The deletion of *PnPf2* diminishes effector expression and effector-triggered susceptibility in wheat carrying *Tsn1* and *Snn3*. (**b**) In addition, PnPf2 functions as a positive regulator of CWDE expression *in planta*. (**c**) Without the full complement of CWDEs being produced, *pf2-69* has a reduced ability to breakdown plant polymers and complex carbohydrates for assimilation needed during *in planta* growth.

We propose a model to explain the role of *PnPf2* during early host infection based on evidence observed in this study (Fig. 6). The removal of PnPf2 drastically diminishes effector expression and so restricts the number of hosts on which *P*. *nodorum* is virulent^9^ (Fig. 6a). Both mutant and wild type strains are able to infect but the reduced ability to produce effectors and cell wall degrading enzymes means that *pf2-69* is delayed in accessing bulk nutrients that come from the early stages of cell necrosis (Fig. 6b). The mutant has reduced access to nutrients stored as complex carbohydrates or compartmentalised in plant cells leading to a reduction in growth during host infection. Increased expression of transporter proteins may be an attempt to scavenge freely available nutrients possibly from the apoplastic space^42^ (Fig. 6c). In addition, we have identified candidate effector genes that are homologous to virulence factors and effectors in other phytopathogens. It is evident that PnPf2 functions to coordinate the expression of a subset of DE genes identified in this study through other TFs. Studies are currently under way to functionally characterise effector candidates and DE TF genes for their role in effector regulation and pathogenicity on wheat.

## Methods

### Infection assays

Whole plant infection assay on two week-old wheat seedlings was performed as previously described^43^. Disease severity was visually determined and scored. A score of zero indicates no disease symptoms. A score of nine indicates a fully necrotised plant. Detached leaf infection assays on two-week old wheat cv. Halberd leaves was performed as previously described^43,44^.

### Biomass analysis using quantitative (Q)-PCR

Q-PCR was to determine fungal biomass from infected wheat. Wheat cv. Halberd was infected with *P*. *nodorum* pycnidiospores as described above. Infection was allowed to develop for three days prior to sampling. The inoculated leaf section was excised and collected. Following this, gDNA was extracted using a Biosprint genomic DNA extraction kit (Qiagen, Venlo, Netherlands). Q-PCR was essentially carried out as described in Brouwer *et al*.^45^ using the primer pair alTubulinqPCRf/r and ActinqPCRhp2F/R (Supplementary Table S3).

### RNA extraction and handling

RNA isolation and *in planta* gene expression analyses were performed as described in Rybak *et al*.^9^ using three day post-infected lesions excised from detached wheat. Library construction and sequencing was performed by the Ramaciotti Centre for Genomics (The University of NSW, Australia). Briefly, the TruSeq Stranded mRNA-seq method was used to prepare all libraries. Following this, sequencing was performed on an Illumina HiSeq. 2500 platform (San Diego, CA, USA) to generate 125 bp paired-end reads. Deep sequencing of all *in planta* samples were carried on individual lanes in the flowcell to ensure maximum sequence data was obtained from low fungal biomass. Samples derived from *in vitro* growth conditions were multiplexed into a single lane. The experiment was performed with four biological replicates.

### RNAseq QC and read trimming

The quality of reads in the FastQ files were assessed using FastQC v0.11.5 (http://www.bioinformatics.babraham.ac.uk/projects/fastqc) before trimming adapter sequences using cutadapt v1.12^46^. Adapter trimmed reads were then filtered into sets belonging to SN15 and wheat using BBSplit v36.67 (https://sourceforge.net/projects/bbmap) using the *P*. *nodorum* genome^16^. Fungal reads were aligned to the SN15 genome using STAR v2.5.0a^47^. Novel splice sites were identified in a first pass alignment of the adapter-trimmed reads of all samples combined. Sample reads were then aligned individually using the novel splice sites identified in the first pass.

### Determining differential gene expression in RNAseq

Fragments overlapping annotated features in the genome were counted using the SubRead featureCount v1.5.1 program using the union mode^48^. Differentially expressed (DE) genes were determined using the R packages EdgeR v3.16.4^49^, DESeq. 2 v1.14.1^50^ and Limma v3.30.6^51^. DE genes were determined from tests of *log*_2_ fold changes (LFC) against the null hypothesis $$-1\le LFC\le 1$$ (i.e. $${H}_{a}=|LFC| > 1$$) using a BH-adjusted *P*-value significance threshold of 0.05. Tests were also performed against the null hypothesis $$LFC\ne 0$$, to be used where greater sensitivity (but lower confidence) was required. Unless otherwise specified, all results refer to thresholded tests ($$|LFC| > 1)$$. Genes that were determined to be DE from tests by all three programs were taken as high-confidence DE sets. For contrasts involving samples with fewer than 10 million fragments (*pf2-69 ip*), genes from these samples were required to have a minimum of 10 CPM to be considered as differentially expressed in the high confidence sets. Normalised FPKM statistics were obtained using Cufflinks v2.2.1^52^. Genes with mean FPKM > 100 were considered to be highly expressed.

### Functional annotation

Functional annotations for existing genes were determined using InterProScan v5.19-58.0^24^. Additionally, carbohydrate active enzymes were predicted using HMMER v3.1b2 (hmmer.org) and dbCAN v5^25^. GO terms^23^ for each gene were found from combined dbCAN and InterProScan results, including matches from: Pfam^53^, TIGRFAM^54^, SMART^55^, PIRSF^56^, PANTHER^57^, HAMAP^58^, Prosite^59^, ProDom^60^, PRINTS^61^, and CATH-Gene3D^62^. Likely protein locations were determined using SignalP v4.1^63^, TargetP v1.1^64^, and TMHMM v2.0c^65^. Predicted proteins with a signal peptide and no transmembrane domains outside of the first 27 amino acids were considered to be secreted. Proteins with effector-like properties were determined using EffectorP v1.0^66^ and were considered to be effector-like if they were also predicted to be secreted using the criteria above. Candidate genes were searched for in SN79-1087 (NCBI, GCA_002267025.1) using Spaln v2.3.3^67^. Overlapping SN79-1087 genes were extracted and protein sequences were aligned using the needle command using EMBOSS^68^.

### Functional enrichment of differentially expressed genes

Over-representation of GO terms in high-confidence differentially expressed gene sets were performed using the R package Goseq v1.26.0^69^. Due to differences in the ability of DESeq. 2, EdgeR, and Limma to handle features with few aligned fragments; enrichment of effector-like or secreted transcripts were determined using the union of differentially expressed genes from all three prediction packages.

### QRT-PCR determination of gene expression

Total RNA extraction from infected wheat cv. Halberd and *P*. *nodorum* mycelia from *in vitro* growth was extracted as described earlier. QRT-PCR was performed using a Quantitect SYBR Green RT-PCR kit (Qiagen, Valencia, CA, USA) and a Bio-Rad (Hercules, CA, USA) CFX96 system. *P*. *nodorum* SN15 gDNA was used as a quantitative standard. The expression value of each gene was normalised against the housekeeping gene actin (*Act1*) using the primer pair ActinqPCRf and ActinqPCRr^70^.

### Analysis of promoters for enriched motifs

Common DNA motifs were discovered from the promoter regions 1.5 kbp upstream (or to the next annotated gene) of predicted transcription start sites of DE genes. Weeder 2.0^71^ was used to search for enriched motifs in these promoters. A full set of SN15 predicted gene promoters was used for background frequencies with the redundancy filter set at 0.5. Utilising the consensus option in MEME v5.0.1^72^, position weight matrices (PWMs) for top non-redundant motifs from each subset were derived for downstream analysis with MEMEsuite tools^73^. Each PWM motif was assessed for overrepresentation in *pf2-69* DE subsets similar to Cho *et al*.^12^. Motif occurrences were first counted using FIMO^74^ and promoters with at least one occurrence were regarded as positive. Significance of over-representation in DE gene promoter sets was determined using Fisher’s exact test with Bonferroni corrected *P*-values (*P*_adj_ < 0.05)^75^ as compared with the full promoter set of SN15. TOMTOM^76^ was used to search the JASPAR NR 2018 databases for matches (E < 1) to published fungal TFBSs in order to characterise the over-represented motifs.

### Y1H assay

The construction of yeast reporter strain and Y1H screening was carried out based on the method of Ouwerkerk and Meijer^77^ with modifications. Y1H bait constructs were prepared by cloning three repeats of the p53 binding site (p53BS) (5′-AGACATGCCT-3′) using the primer pair p53BS-F1/R1^78^, four repeats of the putative *SnToxA* PnPf2 binding site (Pf2BS) (5′-AAGGACCGA-3′) using the primer pair Pf2BS-F1/R1 and four repeats of pf2bs (5′-AAGGAAATA-3′) using the primer pair pf2bs-F1/R1 into pINT1-HIS3NB (provided by Dr. P.B.F Ouwerkerk, Leiden University) (Supplementary Table S3). Repeats of binding sites were cloned into pINT1-HIS3NB. Each construct was linearised, transformed into the yeast strain Y187 (Clontech, CA, USA) and selected on YPAD supplemented with G418. Bait strains were grown on selective media (-His) containing 3-amino-1,2,4-triazole (Sigma-Aldrich, MO, USA). Mating of the yeast bait strains with the prey strains was conducted by mixing the two strains together and grown on YPAD medium. Confirmation of the specific interaction between the bait sequence and the target protein was performed by reintroduce the prey construct into the bait strain. The prey construct pGADT7-p53 was built by cloning partial *p53* from pGBKT7-53 (Clontech, CA, USA) into pGADT7. Similarly, *PnPf2* was amplified from cDNA using the primer pair Pf2-F2/R3 and ligated into pGADT7.

## Supplementary information

SUPPLEMENTARY INFORMATION

Supplementary data S1

Supplementary data S2

Supplementary data S3

Supplementary data S4

Supplementary data S5

Supplementary data S6

Supplementary data S7

## Data Availability

All data generated or analysed during this study are included in this published article (and its Supplementary Information files)
