# Supplementary material for: A specific fungal transcription factor controls effector gene expression and orchestrates the establishment of the necrotrophic pathogen lifestyle on wheat
Source: Sci Rep. 2019 Nov 4;9:15884. doi: 10.1038/s41598-019-52444-7 (PMC6828707; doi:10.1038/s41598-019-52444-7)
Supplement: Supplementary file 1 — SUPPLEMENTARY INFORMATION [file 41598_2019_52444_MOESM1_ESM.docx]

**A specific fungal transcription factor controls effector gene expression and orchestrates the establishment of the necrotrophic pathogen lifestyle on wheat**

Darcy A. B. Jones^1^, Evan John^1^, Kasia Rybak^1^, Huyen T. T. Phan^1^, Karam B. Singh^1,2^, Shao-Yu Lin^3^, Peter S. Solomon^3^, Richard P. Oliver^1^ and Kar-Chun Tan^1^*

^1^School of Molecular and Life Sciences, Centre for Crop and Disease Management, Curtin University, Bentley, 6102, Perth, Australia.

^2^CSIRO Agriculture and Food, Wembley, Western Australia

^3^Division of Plant Sciences, Research School of Biology, The Australian National University, Canberra, ACT, Australia

*Corresponding author

**Email,** [Kar-Chun.Tan@curtin.edu.au](mailto:Kar-Chun.Tan@curtin.edu.au);

**Supplementary Table S1.** A summary of RNAseq fragment read alignments to the SN15 predicted gene set.

| **Treatment** | **Isolate** | **Fragment numbers** | **Fragment numbers aligned to SN15 genome** | **% aligned** | **% aligned fragments intersecting genes** |
| --- | --- | --- | --- | --- | --- |
| *In planta* | *pf2-69* | 3.0E+08 | 3.4E+06 | 1.1 | 91.0 |
| *In planta* | *pf2-69* | 3.0E+08 | 4.6E+06 | 1.6 | 90.7 |
| *In planta* | *pf2-69* | 3.0E+08 | 6.0E+06 | 2.0 | 91.4 |
| *In planta* | *pf2-69* | 3.1E+08 | 5.1E+06 | 1.6 | 91.4 |
| *In planta* | SN15 | 2.5E+08 | 1.8E+07 | 7.3 | 92.2 |
| *In planta* | SN15 | 2.8E+08 | 1.8E+07 | 6.5 | 93.0 |
| *In planta* | SN15 | 2.7E+08 | 1.8E+07 | 6.6 | 93.0 |
| *In planta* | SN15 | 3.0E+08 | 2.2E+07 | 7.4 | 92.9 |
| *In vitro* | *pf2-69* | 2.5E+07 | 2.3E+07 | 93.9 | 92.3 |
| *In vitro* | *pf2-69* | 2.4E+07 | 2.3E+07 | 94.5 | 91.4 |
| *In vitro* | *pf2-69* | 2.3E+07 | 2.1E+07 | 94.3 | 92.3 |
| *In vitro* | *pf2-69* | 2.5E+07 | 2.4E+07 | 92.7 | 91.4 |
| *In vitro* | SN15 | 2.4E+07 | 2.3E+07 | 94.5 | 91.8 |
| *In vitro* | SN15 | 2.5E+07 | 2.4E+07 | 93.8 | 92.1 |
| *In vitro* | SN15 | 2.2E+07 | 2.0E+07 | 93.3 | 91.6 |
| *In vitro* | SN15 | 2.4E+07 | 2.3E+07 | 95.3 | 92.6 |
|  |  |  |  |  |  |

**Supplementary Table S2.** The number of DE genes predicted using EdgeR, DESeq2 and Limma. Genes predicted to be DE by all three tools were taken as a high confidence DE set.

|  | ***ip* vs *iv* SN15** | ***ip* vs *iv***  ***pf2-69*** | ***pf2-69* vs SN15**  ***ip*** | ***pf2-69* vs SN15 *iv*** |
| --- | --- | --- | --- | --- |
| **Up (3 tests)** | 1889 | 1736 | 449 | 323 |
| **Down (3 tests)** | 1393 | 706 | 303 | 342 |
| **DE (<3 tests)** | 162 | 99 | 48 | 9 |
| **Down (<3 tests; *pf2-69* filtered <10 CPM)** | NA | 269 | 163 | NA |

**Supplementary Table S3.** A list of primers used in this study.

**Primer name Sequence (5' to 3') Purpose**

SN30077_qPCRaF TTGTTGGGCAGAGGCAGGGCGG qRT-PCR of SNOG_30077

SN30077_qPCRaR TCTTCTCTCGCACTTGACATCC qRT-PCR of SNOG_30077

ActinqPCRf AGTCGAAGCGTGGTATCCT qRT-PCR of Act1

ActinqPCRf ACTTGGGGTTGATGGGAG qRT-PCR of Act1

qPCR_SNOG_01146F TCTGACGATAAGGCTGGAACC qRT-PCR of SNOG_01146

qPCR_SNOG_01146R CCAGACGATGTAGCAGGTCC qRT-PCR of SNOG_01146

qPCR_SNOG_02755F3 TCAGGGAGGGTTCTTGCAAC qRT-PCR of SNOG_02755

qPCR_SNOG_02755R3 TCCTGAGGCGATGTCATCAC qRT-PCR of SNOG_02755

qPCR_SNOG_08150F1 CGCAGATCCAGGACTACGTC qRT-PCR of SNOG_08150

qPCR_SNOG_08150R1 CGTGTCCTCGGTGTTGGAG qRT-PCR of SNOG_08150

qPCR_SNOG_10736F1 GGCCAAGGCACTCGAAAGAC qRT-PCR of SNOG_10736

qPCR_SNOG_10736R1 TCACTTCGAACCAGTAGGCG qRT-PCR of SNOG_10736

qPCR_SNOG_12350F1 CAGAACGGCAAGACAGGTTG qRT-PCR of SNOG_12350

qPCR_SNOG_12350R1 CTACTCGCTCTTCCTGACC qRT-PCR of SNOG_12350

qPCR_SNOG_14243F1 GGATTCTGCCTCTCGGTGAC qRT-PCR of SNOG_14243

qPCR_SNOG_14243R1 AACACTGTAGCCTCCGGACT qRT-PCR of SNOG_14243

qPCR_SNOG_15270F CTGTATACGGCTGGACTCGC qRT-PCR of SNOG_15270

qPCR_SNOG_15270R ATCGGTCTGGAAGCTACCCT qRT-PCR of SNOG_15270

ToxAqPCRf CGATCCCGGTTACGAAAT qRT-PCR of SnToxA

ToxAqPCRr TTGACATGCAGCTTCCCT qRT-PCR of SnToxA

qPCRSNOG_30352F1 CGCTCGTTCTTATCATGGCC qRT-PCR of SNOG_30352

qPCRSNOG_30352R1 CAGTACCGCCATCGTCTGTT qRT-PCR of SNOG_30352

qPCR_SNOG_30359F CTCTCGATACCCCTGCCAAT qRT-PCR of SNOG_30359

qPCR_SNOG_30359R GCAGAAGAACCTCTCCGCTA qRT-PCR of SNOG_30359

qPCR_13939f3 GCGATGGAGATGTCGTTGAAGG qRT-PCR of SNOG_13939

qPCR_13939r3 ATGCATTTCCAATCCCTCACC qRT-PCR of SNOG_13939

qPCR_02980f1 CGTACGCATGTCAACGAGCATGG qRT-PCR of SNOG_02980

qPCR_02980r1 GGCCTTGATAGCTGCGCTCAACG qRT-PCR of SNOG_02980

SN30077_qPCRaF TTGTTGGGCAGAGGCAGGGCGG qRT-PCR of SNOG_30077

SN30077_qPCRaR TCTTCTCTCGCACTTGACATCC qRT-PCR of SNOG_30077

alTubulinqPCRf CGAGGAAACCTTGCAGACCCG Biomass assay using genomic DNA

alTubulinqPCRr CACCCGGAGCACATGATCACC Biomass assay using genomic DNA

ActinqPCRhp2F GGAAGGACCGCTCTCGTCGTAC Biomass assay using genomic DNA

ActinqPCRhp2R GCCCCGTCGTCCATGAAGGTC Biomass assay using genomic DNA

Pf2BS-F1 GGCCGCAAGGACCGAAAGGACCGAAAGGACCGAAAGGACCGAA Y1H

Pf2BS-R1 CTAGTTCGGTCCTTTCGGTCCTTTCGGTCCTTTCGGTCCTTGC Y1H

pf2bs-F1 GGCCGCAAGGAAATAAAGGAAATAAAGGAAATAAAGGAAATAA Y1H

pf2bs-R1 CTAGTTATTTCCTTTATTTCCTTTATTTCCTTTATTTCCTTGC Y1H

p53BS-F1 GGCCGCAGACATGCCTAGACATGCCTAGACATGCCTA Y1H

p53BS-R1 CTAGTAGGCATGTCTAGGCATGTCTAGGCATGTCTGC Y1H

Pf2-F2 CGACGTACCAGATTACGCTCATATGTCGTCCAGCAGTACC Y1H

Pf2-R3 TCGATGCCCACCCGGGTGGAATTCATTGCTTGAACATCATAGATG Y1H


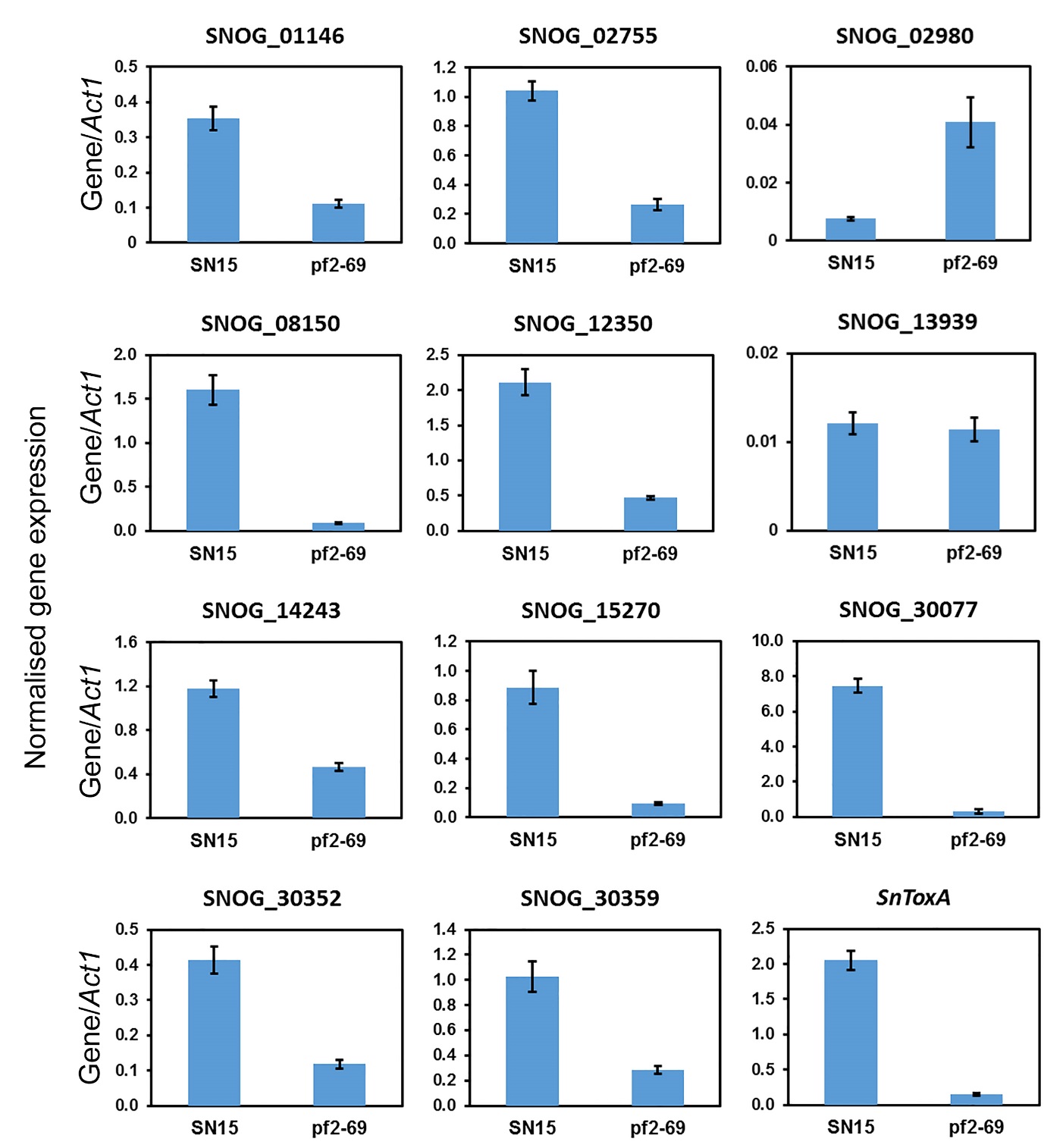


**Supplementary Fig S1.** Quantitative RT-PCR analysis of PnPf2-regulated candidate effector gene expression in SN15 and *pf2-69* at three days post infection on wheat cv. Halberd. The expression of SNOG_10736 was not detected. Standard error bars are shown (*n* = 3).


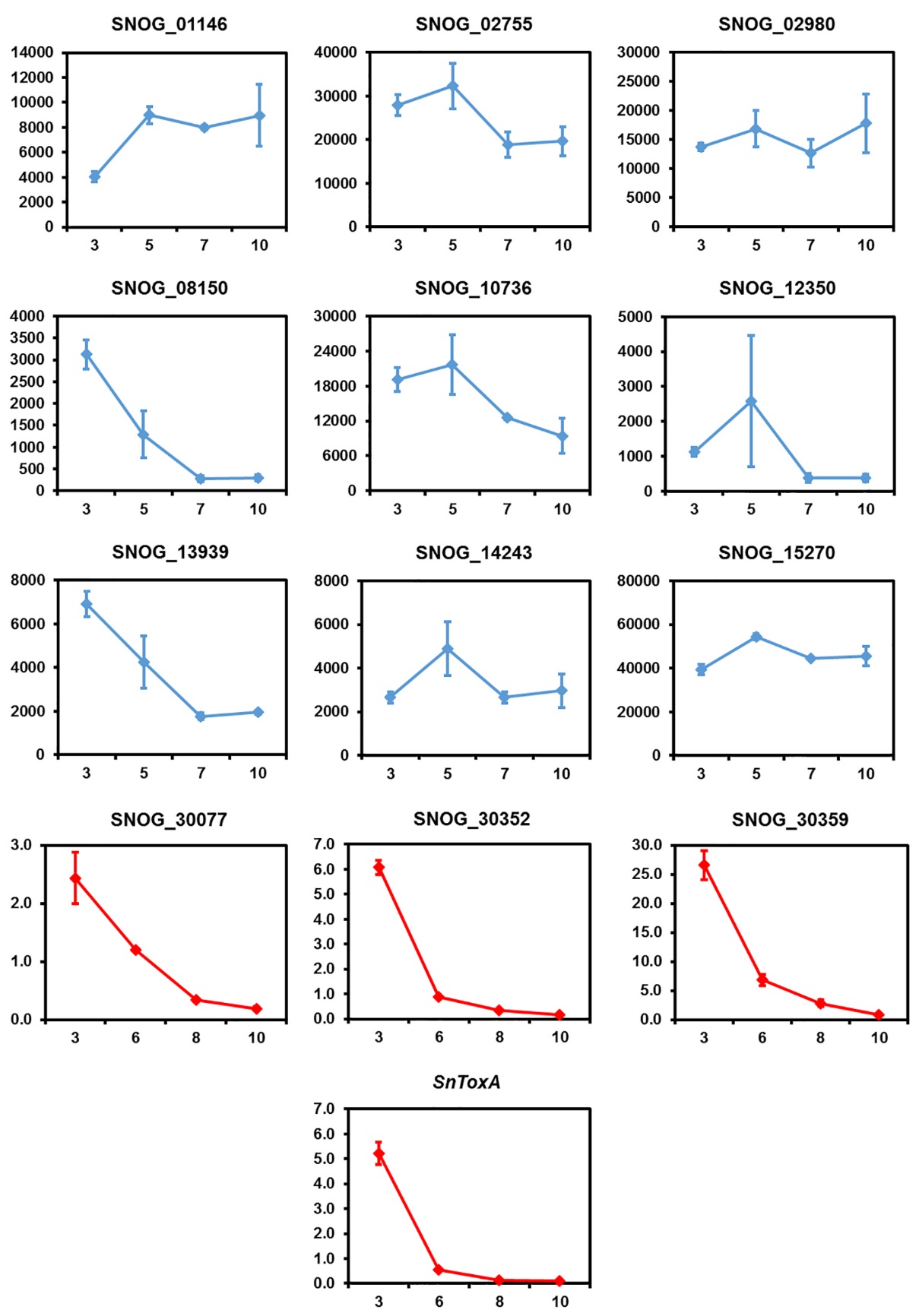


**Supplementary Fig S2.** Gene expression profile of PnPf2-regulated candidate effector genes. The *x*-axis represent days post-infection. Gene expression derived from previous microarray data (blue)^18^ and qRT-PCR (red)^9 for SnToxA; This study^. Relative expression for microarray and expression relative to *Act1* for qRT-PCR are indicated on the *y*-axis. Standard error bars are shown (*n* = 3).


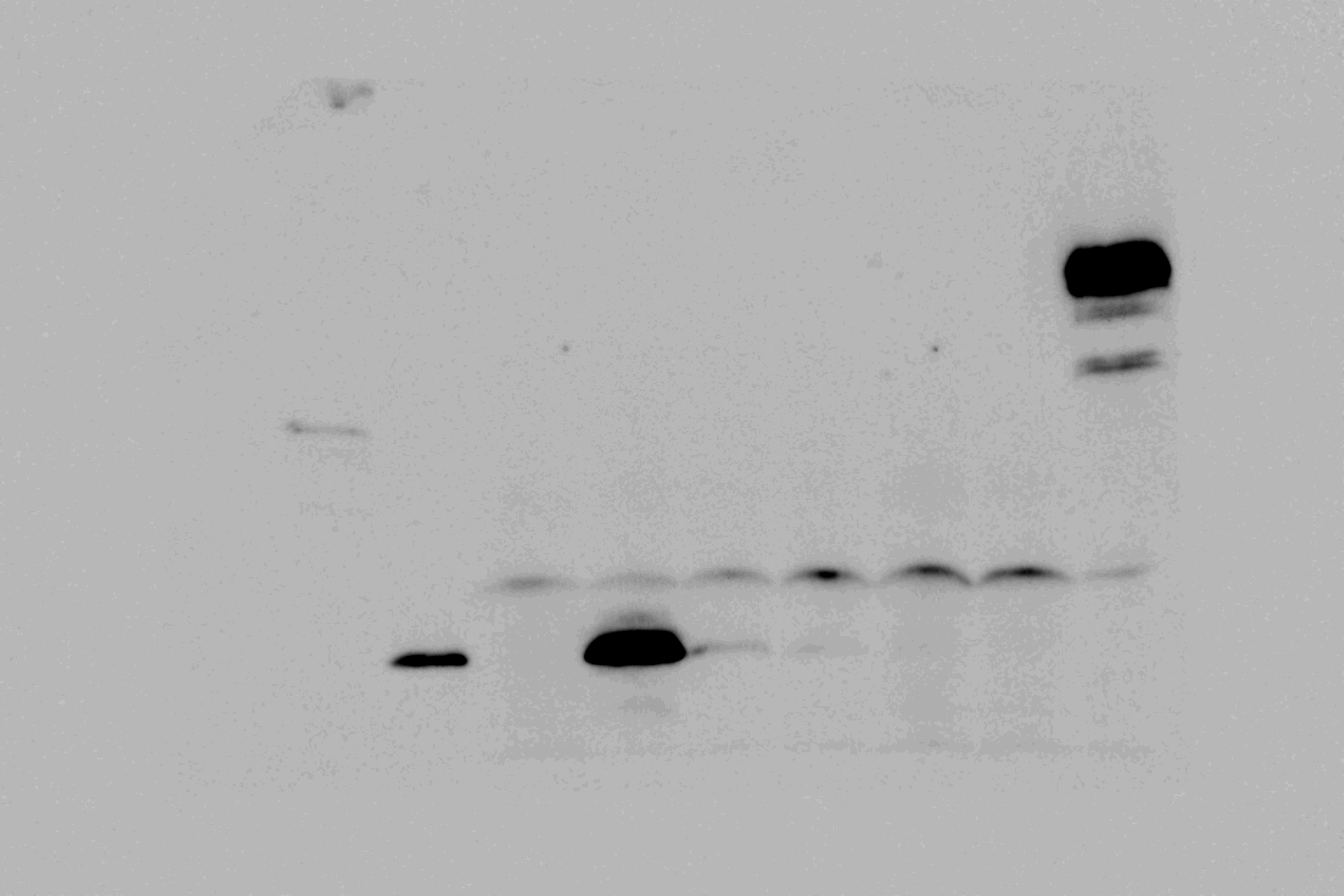


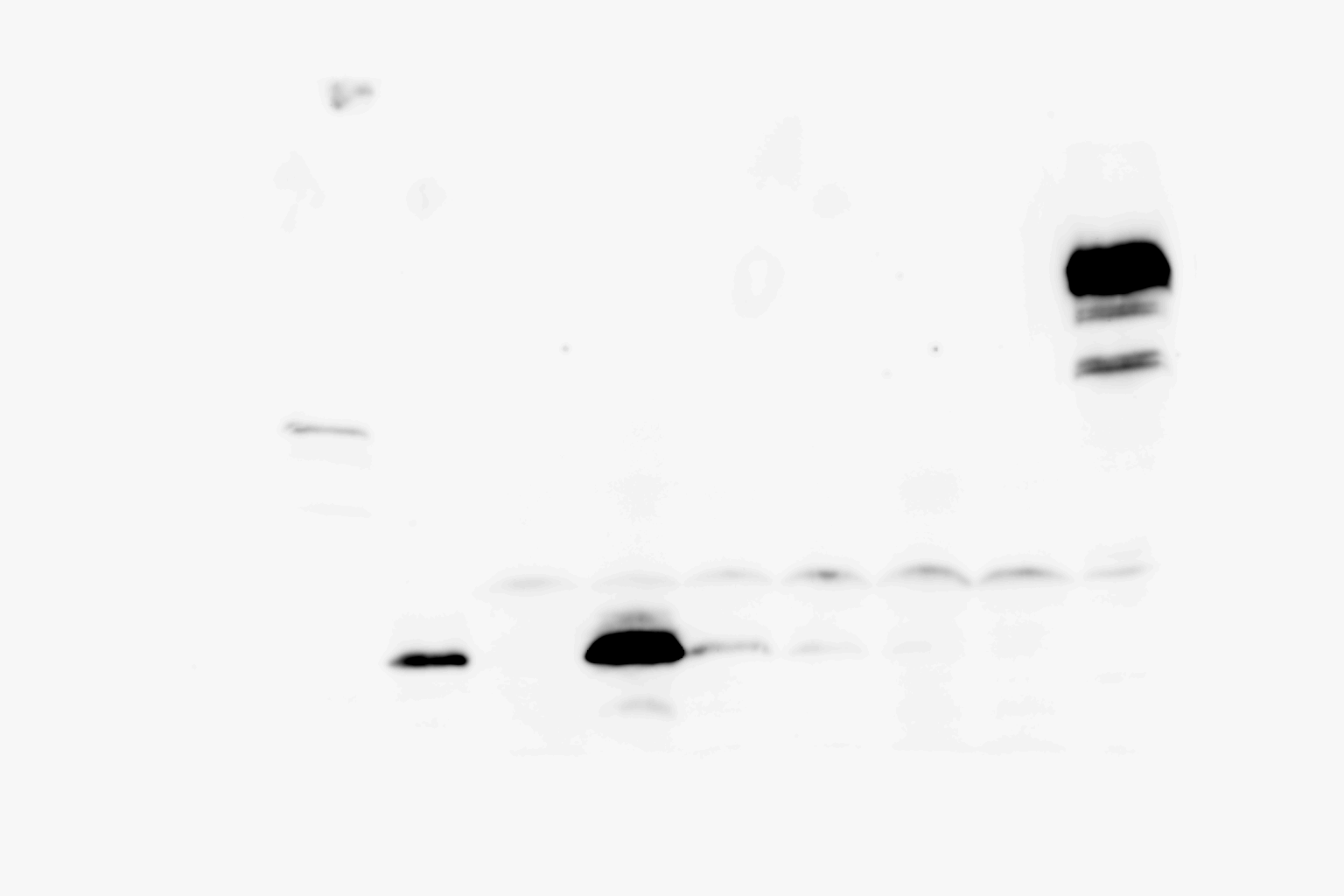


**Supplementary Fig S3.** Original gel photos for the top left panel of Fig 5b. *Top panel*, Unedited photo. *Bottom panel*, Contrast-adjusted photo. Red box indicate cropping. Original photos for other cropped panels cannot be located.

**Supplementary dataset captions**

**Supplementary Data S1.** Counts of RNAseq read pairs aligned to previously published *P. nodorum* SN15 genes^16^. Sheet “Feature Counts” is the output from SubRead’s featureCount command showing the raw counts for each gene. The sheet “Normalised Counts” contains normalised variance stabilising transformed (VST) counts from DESeq2, which are more suitable for comparison between treatments. The sheet “FPKMs” contains normalised fragments per kilobase per million (FPKM) counts from Cufflinks software.

**Supplementary Data S2.** A summary of principal component (PC) analyses of normalised RNAseq fragment counts for SN15 and *pf2-69* *P. nodorum* isolates *in planta* and *in vitro*. Loadings values for genes in PC1 and PC2 are presented alongside functional annotations.

**Supplementary Data S3.** Summary tables combining results from all DE tests and functional annotations. The sheet “Summary” contains transcript lengths, mean fragment (raw) counts, mean log_2_ fold changes (LFCs), results from DE tests (up, down, or same [i.e. not DE]), and results from key functional annotation software/databases. Where a gene has multiple functional annotations from the same resource, each term is collapsed into a single cell delimited by a semicolon (;). The sheet “GO terms” is similar to “Summary” but only GO functional annotations are included, and multiple terms are provided on separate rows (duplicating the expression data). This is provided to make grouping genes with the same GO terms easier using simple column sorting.

**Supplementary Data S4.** Functional annotations of previously published *P. nodorum* SN15 putative proteins. Sheets summarise results from InterProScan, HMMER matches to dbCAN, SignalP, TargetP, TMHMM, EffectorP, LOCALIZER, and BLAST matches to effector candidates in PHIbase. The tables “Panther families” and “Superfamilies” are based on information in “InterProScan”, but are supplemented with additional information not provided in the InterProScan results. Gene ontology (GO) term associations are based on dbCAN and InterProScan results, excluding SUPERFAMILY matches.

**Supplementary Data S5.** Results of GO enrichment analyses the differentially expressed genes. There are separate sheets for each contrast comparing *P. nodorum* isolates SN15 and *pf2-69*, *in planta* (*ip*) and *in vitro* (*iv*). Three enrichment tests were performed per GO term (“category” column) indicated in the “direction” column, for up-regulated genes (denoted “up”), down-regulated genes (denoted “down”), and up- or down-regulated genes (denoted “diff”). Columns “number_in_cat” and “number_de_in_cat” indicate the number of genes with the GO term, and the number of those genes that are differentially expressed in the direction indicated by the “direction” column.

**Supplementary Data S6.** HTML descriptions of *pf2-69* vs SN15 GO enrichment and network plots with detailed labels. (a) *pf2-69* vs SN15 *ip* GO network. (b) *pf2-69* vs SN15 *ip* GO representation. (c) *pf2-69* vs SN15 *iv* GO network. (d) *pf2-69* vs SN15 *iv* GO representation.

**Supplementary Data S7.** A summary of motif frequency from DE gene promoters.
